# Lupus vulgaris revealed by chronic nasal ulceration

**DOI:** 10.11604/pamj.2024.47.75.42491

**Published:** 2024-02-20

**Authors:** Anjana Ledwani, Ashwin Karnan

**Affiliations:** 1 Department of Respiratory Medicine, Jawaharlal Nehru Medical College, Datta Meghe Institute of Higher Education and Research, Sawangi (Meghe), Wardha, Maharashtra, India

**Keywords:** Skin tuberculosis, erythema, granuloma, tuberculin

## Image in medicine

A 40-year-old male presented to the outpatient department with complaints of itching and ulceration around the nose for the past 1 year. On examination, the patient was conscious, oriented, vitally stable, and had an erythematous plaque with crusting and loss of hair around the nose with bilateral micro nares with a depressed nasal bridge and deviated nasal septum to the left side. The patient had no significant past, personal, and family history and had no history of any addictions. Relevant investigations were done, the erythrocyte sedimentation rate (ESR) was raised (110mm/hr), and the Mantoux test was done which showed an induration of 30mm which was strongly positive for tuberculosis. Chest X-ray was within normal limits and sputum for acid-fast bacilli was negative. An incisional biopsy was done from the lesion over the nose, which showed caseating granuloma with epithelioid cells and Langhans giant cells along with lymphocytes and plasma cells suggestive of lupus vulgaris. The patient was started on anti-tubercular treatment consisting of a fixed-dose combination of isoniazid, Rifampicin, Pyrazinamide, and Ethambutol according to the weight band. The patient is on regular follow-up and is showing signs of clinical improvement. Lupus vulgaris is the most common type of skin tuberculosis. It is also known as Tuberculosis Cutis Luposa. It occurs due to the chronic infection of *Mycobacterium tuberculosis* and is transmitted by hematogenous and lymphatic spread. The contiguous extension is the most common. It mainly affects the head and neck region. Diagnosis is done by a histopathological finding of tuberculoid granuloma with lymphocytes, plasma cells, central caseation, epithelioid cells, and giant cells. The treatment comprises anti-tubercular drugs for 6-9 months.

**Figure 1:**
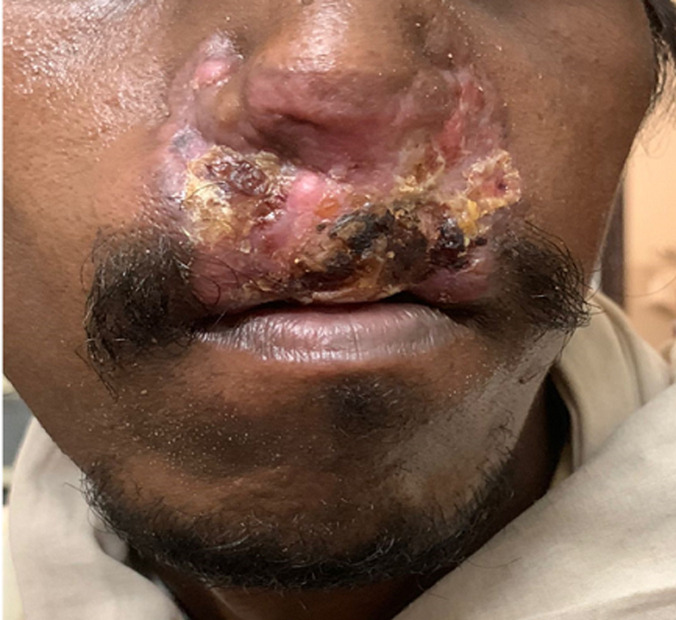
erythematous plaque with crusting and loss of hair around nose and philtrum with destruction of nares

